# Engaging communities, modeling systems: lessons from system dynamics modeling on maternal health in Texas

**DOI:** 10.3389/fgwh.2025.1577568

**Published:** 2025-09-25

**Authors:** Kyrah K. Brown, Michael K. Lemke, Deneen Robinson, Saeideh Fallah-Fini, David W. Lounsbury, Thanayi Lambert, Mercy J. Obasanya, Tiffany B. Kindratt

**Affiliations:** 1 Department of Kinesiology, The University of Texas at Arlington, Arlington, TX>, United States; 2 Department of Environmental and Occupational Health Sciences, University of Texas Health Science Center at Houston School of Public Health, San Antonio, TX, United States; 3 Truth Pregnancy Resource Center, Dallas, TX, United States; 4 Department of Industrial and Manufacturing Engineering, California State Polytechnic University, Pomona, Pomona, CA, United States; 5 Department of Epidemiology and Population Health, Albert Einstein College of Medicine, Bronx, NY, United States; 6 Department of Family and Social Medicine, Albert Einstein College of Medicine, Bronx, NY, United States

**Keywords:** severe maternal morbidity, maternal mortality, system dynamics modeling, group model building, community-based participatory research, reproductive health

## Abstract

**Introduction:**

Disparate maternal health outcomes among non-Hispanic Black women stem from intricate, interrelated factors shaped by clinical, social, and structural influences. Traditional approaches often fall short in addressing these complexities, necessitating a shift toward systems thinking and community-driven solutions.

**Methods and materials:**

This paper describes the lessons learned from the implementation of system dynamics group model building (SD GMB) workshops grounded in community-based participatory research (CBPR) principles in two separate projects focused on maternal health among Black women. We recruited 31 diverse stakeholders, including individuals with lived experience, and applied trauma-informed facilitation, wraparound support, and structured systems modeling activities. A descriptive analysis of workshop data was performed to accompany the identified lessons learned.

**Results:**

Evaluation data from surveys and open-ended responses indicated high stakeholder satisfaction, increased capacity to apply systems thinking, and a shift from skepticism to agency. Stakeholders valued the inclusive design, reported meaningful learning, and expressed interest in future engagement. The workshops fostered transformative learning and generated actionable systems insights rooted in community experience.

**Discussion:**

This work demonstrates how SD GMB, when integrated with CBPR, can build trust, elevate marginalized voices, and produce models that reflect structural realities. Future directions include quantifying the models, hosting learning labs to test interventions, and developing an open-access dashboard to simulate policy scenarios. These findings contribute to ongoing efforts to design more engaging, community-informed approaches to maternal health research and practice.

## Introduction

Globally, maternal health outcomes are widely recognized as indicators of a nation's overall health. In 2022, the United States reported 22 maternal deaths per 100,000 live births—two to three times higher than the rates observed in other high-income countries (1). Maternal mortality, however, is only the tip of a broader public health crisis. For every maternal death, 50–100 women experience severe maternal morbidity (SMM)—life-threatening conditions that often contribute to mortality or long-term disability (2). SMM is a key risk factor for maternal mortality as it involves conditions that can result in maternal death if left unaddressed. SMM encompasses a range of complications, including obstetric hemorrhage, organ system failure, stroke, and other physical or mental health conditions (2). However, the burden of SMM is not equally distributed. Non-Hispanic Black women face an SMM rate two times higher than the national rate (2) and are also disproportionately affected by cardiovascular severe maternal morbidity (CSMM), which includes cardiac arrest, myocardial infarction, heart failure, and stroke (3, 4).

Adverse maternal health outcomes emerge from complex, interdependent systems characterized by clinical, social, and structural influences (5–8). Yet, dominant frameworks remain reductionist and ill-equipped to address the dynamic complexity of these systems (9). Instead, maternal and child health research has often focused on individual risk factors—such as late prenatal care or obesity—while giving less attention to the systemic conditions that shape them (7, 10). This narrow lens also limits intervention strategies, which are frequently tested in isolation and confined to clinical settings (11–13). Thus, addressing the root causes of SMM and reducing maternal mortality requires a shift toward systems thinking, grounded in both conceptual and methodological innovation.

Complex systems science approaches—especially system dynamics modeling—offer valuable tools for understanding the multifaceted drivers of maternal health outcomes. While system dynamics modeling has gained traction in broader public health domains (14–16), its application in maternal health remains limited. In the United States, few studies have used system dynamics modeling to examine disparate reproductive health outcomes (9, 17–19), despite its potential to inform transformative solutions. System dynamics modeling is particularly effective when paired with participatory approaches. One such method, system dynamics group model building (SD GMB), engages stakeholders in co-developing models to foster shared understanding, build consensus, enhance team learning, and align stakeholders around effective intervention strategies. Tools such as causal loop diagrams (CLDs) and stock-and-flow diagrams help visualize feedback mechanisms and dynamic relationships that shape public health problems such as adverse maternal health outcomes (20). However, SD GMB offers more than a technical framework for modeling—it provides a collaborative process that supports shared learning and inclusive dialogue (21). Aligned with theories of transformative learning, SD GMB enables stakeholders to move from passive involvement to active co-creation of knowledge and solutions (22). Through structured reflection and group sense-making, the process encourages new ways of thinking and fosters a sense of shared responsibility—both essential elements for addressing complex public health challenges. Moreover, this approach is relevant to maternal health research because it allows for the integration of diverse perspectives, including those of Black women who have been historically excluded in research and policy discussions (23). By engaging community stakeholders directly in the modeling process, SD GMB helps illuminate the clinical, social, and structural factors that shape adverse maternal health outcomes (9). It also builds systems thinking capacity and supports the development of context-specific insights that can inform more responsive and sustainable solutions.

There is limited literature on the use of participatory approaches in system dynamics modeling to address maternal health disparities. This paper aims to (1) describe our approach to engaging community stakeholders in SD GMB workshops, guided by community-based participatory research (CBPR) principles (24), and (2) share lessons learned, supported by evaluation data from the workshops. This work contributes to the growing effort to make system dynamics and systems thinking more accessible to non-technical audiences by illustrating how community stakeholders can be actively involved in modeling processes (17, 19, 20, 25, 26). In doing so, this paper advances the implementation and dissemination of systems thinking and system dynamics in maternal health disparities research.

## Methods

### Team background

Our research team includes investigators with complementary experience and expertise in maternal and reproductive health, community psychology, complex systems science in public and population health, system dynamics modeling, clinical care, and large-scale secondary data analysis. One team member serves as a community investigator/consultant and has expertise in health policy, maternal and child health services, and reproductive justice. This team member previously participated in our pilot system dynamics group model building project and joined this work to continue advancing community-centered research. Our team also includes consultants with expertise in participatory system dynamics and technical modeling, as well as undergraduate and graduate students who support research and engagement activities. Several of us have worked together on prior projects, building a foundation of trust and collaboration. Some of us have long-standing relationships with local organizations and communities, particularly those serving Black women in Texas, which have strengthened our ability to conduct research that is both contextually grounded and responsive to community needs.

### Project background

In 2023, our research team received two separate grant awards to implement distinct projects that apply complex systems science approaches to maternal health disparities among non-Hispanic Black women. For the purposes of this paper, we refer to these efforts collectively as *Project IMPACT* (**I**mproving **M**aternal and re**P**roductive he**A**lth using **C**omplex systems simulation **T**echniques) to describe shared lessons learned across both initiatives.

Although both projects center on maternal health among Black women, they differ in scope, problem definition, and geographic focus. The first project investigates SMM among non-Hispanic Black women in Texas—a state that ranks last nationally in healthcare access and affordability (27) and where the Black SMM rate is twice that of the general population (28). The second project, which is part of a national research collaborative, focuses specifically on cardiovascular-related SMM among non-Hispanic Black women in the Dallas–Fort Worth (DFW) metroplex—the fourth largest metropolitan area in the United States (29) and a leading contributor to maternal deaths among this population (28, 30). By combining insights from these two distinct but complementary projects, this paper offers a broader perspective on how participatory system dynamics approaches can be applied across different contexts to address maternal health inequities.

### Stakeholder recruitment

Our team used the same stakeholder recruitment process for both projects. First, we generated a list of potential stakeholders that had specialized knowledge in severe maternal morbidity or cardiovascular severe maternal morbidity as clinical outcomes, as well as other domains known to shape maternal health such as policy, sociohistorical forces, life course, neighborhood forces, criminal justice, and social services. We identified potential stakeholders based on existing relationships, referrals from existing community partners, and targeted web searches. Our recruitment process prioritized stakeholder heterogeneity by engaging “unusual suspects,” such as community historians, housing officials, environmental health advocates, and disability rights professionals. We generated a list of 95 potential stakeholders that we considered for either of the two projects (depending on their expertise). We narrowed down the initial list using a power-versus-interest matrix for each project to ensure inclusion of diverse perspectives, and selection was based on relevance of expertise, influence, and interest in the problem (31). We identified and ranked 69 potential individuals or organizations for recruitment (36 for the SMM Project and 33 for the CSMM Project). Of these stakeholders, 35 agreed to participate, though 3 later withdrew due to scheduling conflicts. In total, 32 stakeholders completed the workshops—17 in the SMM Project and 15 in the CSMM Project. Three stakeholders also participated in our 2021 pilot SD GMB project on maternal mortality: two in the SMM Project and one in the CSMM Project.

Overall, most of the participating stakeholders were women (93.8%), non-Hispanic Black (81.3%), and 30–49 years old (68.8%). Thirty-one percent of the stakeholders self-reported lived experience, meaning that they personally experienced (or had a partner or family member experience) SMM or CSMM. Among the stakeholders who represented an organization or agency, 40% worked at a Black-led organization and 57% worked at a woman-led organization. Three stakeholders participated as unaffiliated community members. Table 1 presents the demographic characteristics of the participating stakeholders across both projects.

**Table 1:** Self-reported demographic characteristics of the community stakeholders by project.

Project	Lived experience	Ethnicity and race	Gender identity	Role description	Sector	Black-led organization	Woman-led organization
Project IMPACT: SMM	No	NH Black	Woman	Government housing agency professional	Government (including governmental public health)	No	No
Project IMPACT: SMM	No	NH Black	Woman	Disability rights attorney	Non-profit or grassroots	No	No
Project IMPACT: SMM	No	NH Black	Man	Child development specialist and community health worker	Non-profit or grassroots	No	No
Project IMPACT: SMM	No	NH Black	Woman	Research Scientist	Healthcare/ medical	No	Yes
Project IMPACT: SMM	No	NH Black	Woman	MCH program manager; local health department	Government (including governmental public health)	No	No
Project IMPACT: SMM	No	NH Black	Woman	Labor and delivery nurse	Healthcare/ medical	No	No
Project IMPACT: SMM	Yes	NH Black	Man	Father and community health worker	Non-profit or grassroots	Not applicable	Not applicable
Project IMPACT: SMM	Yes	NH Black	Woman	Research scientist and health educator	Non-profit or grassroots	Yes	Yes
Project IMPACT: SMM	Yes	NH Black/multi-racial	Non-binary	Director of community-based organization	Non-profit or grassroots	Yes	Yes
Project IMPACT: SMM	No	NH Black	Woman	Reproductive justice organization research analyst	Non-profit or grassroots	Yes	Yes
Project IMPACT: SMM	Yes	NH Black	Woman	Community historian and community member	N/A	Not applicable	Not applicable
Project IMPACT: SMM	No	NH White	Woman	Researcher/professor	Academia/ research	No	No
Project IMPACT: SMM	No	NH Black	Woman	Environmental health commissioner	Government (including governmental public health)	No	No
Project IMPACT: SMM	No	NH Black	Woman	Certified nurse midwife	Healthcare/ medical	Yes	Yes
Project IMPACT: SMM	Yes	NH Black	Woman	Reproductive justice program coordinator and full-spectrum doula	Non-profit or grassroots	Yes	Yes
Project IMPACT: SMM	No	NH White	Woman	Community consultant and business owner with expertise in maternal health	Private/business	No	Yes
Project IMPACT: SMM	Yes	NH Black	Woman	Program director for HIV/AIDS; reproductive justice organization	Non-profit or grassroots	Yes	Yes
Project IMPACT: CSMM	No	NH Black	Woman	Government housing official	Government (including governmental public health)	No	No
Project IMPACT: CSMM	No	NH Black	Woman	Cardio-obstetrician	Healthcare/ medical	No	No
Project IMPACT: CSMM	Yes	NH Black	Woman	Postpartum cardiomyopathy advocate and community-based organization founder	Non-profit or grassroots	Yes	Yes
Project IMPACT: CSMM	No	NH Black	Woman	Obstetrician/gynecologist and chief medical officer	Healthcare/medical	No	No
Project IMPACT: CSMM	No	NH Black	Woman	Certified professional midwife at a community-based midwifery practice	Healthcare/medical	Yes	Yes
Project IMPACT: CSMM	No	NH Black	Woman	Licensed midwife at a community-based organization	Healthcare/medical	Yes	Yes
Project IMPACT: CSMM	No	NH Black	Woman	Birth doula; doula trainer; and business owner	Non-profit or grassroots	Yes	Yes
Project IMPACT: CSMM	No	NH Black	Woman	Registered nurse	Other: community-based public health nurse	No	No
Project IMPACT: CSMM	Yes	NH Black	Woman	Health department division director	Government (including governmental public health)	No	No
Project IMPACT: CSMM	No	NH Black	Woman	Postpartum doula; doula trainer; and organization founder	Non-profit or grassroots	Yes	Yes
Project IMPACT: CSMM	Yes	NH Black	Woman	Certified professional midwife	Healthcare/medical	Yes	Yes
Project IMPACT: CSMM	Yes	NH Black	Woman	Mother; postpartum cardiomyopathy advocate	Other: community advocacy	Yes	Yes
Project IMPACT: CSMM	No	NH White	Woman	Research scientist at healthcare institution	Academia/research	No	No
Project IMPACT: CSMM	Yes	NH Black	Woman	Mother; community advocate	Other: community advocacy	Not Applicable	Not applicable
Project IMPACT: CSMM	No	NH Black	Woman	Director of maternal and child health program	Government (including governmental public health)	No	No

MCH, maternal and child health; NH, non-Hispanic.

### System dynamics group model building workshop planning and implementation

Our team spent 3 months designing the SD GMB workshops, drawing on best practices from the literature (32, 33) and lessons from prior experiences (17, 25, 34). This included attention to technical elements such as problem definition and engaging stakeholders with mental models, as well as trust-building exercises, fostering co-learning through stakeholder interaction, and thoughtfully incorporating participant input—such as accessibility needs—into the workshop design.

Each project included two SD GMB workshop sessions held in March 2024, spaced 2 weeks apart. The first session spanned a full day and a half (Friday and Saturday), while the second session lasted one full day (Friday). The first project (Project IMPACT: SMM) was held during the first and third weeks of the month, and the second project (Project IMPACT: CSMM) was held during the second and fourth weeks. We intentionally grounded our workshop design in core CBPR principles (24, 33) and other community-centered approaches. Table 2 outlines how our strategies aligned with CBPR values. The workshops were held at a public university centrally located in the DFW metroplex. We selected a building that allowed us to reserve a large classroom, two breakout rooms, a wellness room, and an on-site childcare space—all on the same floor. The building exceeded Americans with Disabilities Act accessibility standards and was located directly next to a visitor parking garage requiring minimal signage for navigation. To reduce logistical burdens associated with university campuses, we covered stakeholders' parking costs and used the university's digital pass system.

**Table 2:** Alignment of project approach with CBPR principles.

CBPR principle	Examples from the project
Recognizes community as a unit of identity	• Engaged stakeholders with lived experience and community-based roles • Included diverse community stakeholders such as housing officials and environmental health advocates
Builds on strengths and resources within the community	• Leveraged existing relationships and referrals from community partners • Emphasized the advances to maternal health that the stakeholders have already made prior to the project.
Facilitates collaborative partnerships in all phases of the research	• Stakeholders co-created community agreements and participated in iterative model building • Conducted member-checking meetings and incorporated stakeholder feedback into final outputs.
Integrates knowledge and action for the mutual benefit of all partners	• Provided stakeholders with tools and training to apply systems thinking in their own work • Shared modeling outputs and project updates through interactive emails and webinars
Promotes a co-learning and empowering process	• Hosted orientation sessions and provided educational materials to build capacity • Encouraged stakeholders to teach-back causal loop diagrams during workshops
Involves a cyclical and iterative process	• Held two workshop sessions with iterative refinement of models between sessions • Used monthly updates and asynchronous feedback tools to maintain engagement
Addresses health from both positive and ecological perspectives	• Included stakeholders from various domains influencing maternal health • Used the 5 R's and BOTGs to identify system variables and visualize trends
Disseminates findings and knowledge gained to all partners	• Sent summaries of workshop activities and modeling outputs via email • Shared manuscript drafts with stakeholders for review and acknowledgment preferences • Produced a project summary video
Involves long-term commitment to process and sustainability	• Maintained ongoing communication through monthly emails and follow-up meetings • Provided stipends, childcare, transportation, and lodging to support sustained participation • Used monthly updates and asynchronous feedback tools to maintain engagement

Understanding that many stakeholders—particularly those with lived experience or community-based roles—might face challenges in attending, we implemented several measures to reduce participation barriers. We provided cash stipends of $25 per hour for up to 35  hours of participation^1^, opting for cash over gift cards to offer greater autonomy over how to use their compensation. We also offered free on-site childcare, transportation via rideshare apps, meals throughout the day, and lodging for those traveling from out of town.

In preparation for the workshops, we prioritized stakeholder orientation and capacity building. Drawing from our pilot work, we recognized the importance of clearly communicating stakeholders’ roles as experts and the overall goals and end-products of the SD GMB process as early and frequently as possible. Two weeks before the workshops, we hosted and recorded virtual orientation sessions that introduced the project, outlined stakeholder expectations, and previewed planned activities. We supplemented these sessions with videos of other SD GMB workshops [e.g., Social System Design Lab (35)] and sent “know before you go” emails using email marketing software [e.g., Mailchimp (36)]. These emails included the orientation recording, workshop examples, and logistical information.

To further support learning, each stakeholder received a personalized binder containing workshop materials, Texas maternal health data reports, a glossary of key terms, and a set of Habits of a Systems Thinker flashcards (37). These resources were designed to build systems thinking capacity and encourage stakeholders to apply these tools in their own work. Recognizing the emotional weight of topics such as SMM, we incorporated structured breaks, mindfulness exercises, and access to a meditation room to support psychological well-being throughout the workshops. To support relationship and trust building among stakeholders and the team, we facilitated formal and informal introductions during breaks, encouraged stakeholders to talk to someone they did not know during breaks, and included a stakeholder contact list in each binder.

In the following section, we briefly describe the SD GMB workshop activities. A detailed technical account of procedures is beyond the scope of this paper and is published separately alongside formal workshop outputs (43).

Prior to the first session, the research team prepared agendas, facilitation plans, and materials. After introducing the project, stakeholders co-created community agreements, which were revisited at the start of each session. We then facilitated a *Concerns and Hopes* activity, adapted from Scriptapedia's *Hopes and Fears*, to promote psychological safety and transparency (38). We collected sticky notes with the stakeholders' hopes and concerns and thematically organized them in real time on a wall. We then engaged in a candid discussion about their concerns and hopes. While stakeholders expressed their concerns, we were careful to practice active listening and avoid responding to their concerns during sharing time. Overall, this activity helped us establish a benchmark for expectations which we revisited during closing reflections. Next, stakeholders engaged in scripted activities using the *5 R's* framework—results, roles, relationships, rules, and resources—to identify system variables through individual brainstorming and group clustering (39). Stakeholders then created behavior-over-time graphs (BOTGs) to visualize trends and key turning points, informing the iterative co-creation of CLDs in small groups. The session ended with a model review and reflection. Between sessions, the team analyzed Workshop 1 data, including thematic coding of the 5 R's, BOTGs, and CLDs, refining the qualitative model and identifying gaps. In Workshop 2, stakeholders worked in small groups to refine their CLDs and identify additional data sources, key individuals, publications, and interpretive considerations. The iterative process kept stakeholder insights central and included coaching to enhance their ability to teach back the CLDs. Each team presented its CLD to the full group during the final session.

Immediately after the SD GMB workshop, we sent interactive emails via our email marketing software to summarize the key workshop activities, corresponding modeling outputs, and how these outputs will inform next steps. To promote transparency and support continued learning, we sent monthly emails with project updates, links to tools we built to facilitate asynchronous feedback, and links to ongoing webinars and trainings. Four months after the workshops, we conducted virtual member-checking meetings, incorporating stakeholder feedback to refine CLDs. We also shared drafts of manuscripts focused on modeling outputs for stakeholders to review and indicate acknowledgment preferences.

### Evaluation measures and data analysis

Prior to the workshop, stakeholders completed a 56-item registration and baseline survey via QuestionPro (40), which asked questions for workshop planning and logistics, and about demographics and organizational characteristics, and baseline knowledge of systems science and group model building (see Supplementary File 1).

We administered a post-session survey for stakeholders to complete at the end of the first full day and a half day during workshop Session 1. Most of the question items were adapted from an original instrument developed by Zimmerman et al. (25) (see Supplementary File 1), including questions that stakeholders rated on a scale (“very good” to “very poor”), such as “the overall quality of today's session was…,” “the degree to which group members” ideas were understood and acknowledged during today's session was…,” and “at the present time, your understanding of the model building process is….” The survey also featured open-ended questions such as “after today's session, my biggest concern about the modeling process is….”

We administered a final evaluation survey at the end of the second workshop session (Supplementary File 1). This survey included the 17 post-session question items and an additional 49 question items focused on evaluating their participation in the system dynamics group model building workshops. This survey included question items about stakeholders’ satisfaction with the project, satisfaction with compensation and other supports, intentions to apply their knowledge to their own work, perceptions about whether the project was a good use of their time, and interest in engaging in future work with the research team. The survey also included questions adopted from instruments developed by Zimmerman et al. (25, 26). Key measures included a 10-item facilitator quality scale designed to measure the quality of the facilitator team and their engagement with stakeholders (Likert scale from 1 “strongly agree” to 5 “strongly disagree”) and 5-item capacity building scale (*α* = 0.90) designed to measure the extent to which stakeholders felt their participation increased their capacity to advance their own work (Likert scale from 1 “not at all” to 5 “to a very great extent”) (25, 26).

We used IBM SPSS to calculate frequencies and percentages to describe the demographic and organizational characteristics of the participating stakeholders across both projects. We calculated means and standard deviations for continuous variables such as the facilitator quality scale and capacity building scale and compared the means and deviations of stakeholders with and without lived experience. Finally, we extracted stakeholders' responses from an open-ended question (e.g., “after today's session, my biggest concern about the modeling process is…”) on the two post-session surveys and the final survey to assess how stakeholder concerns shifted over time.

## Results: lessons learned from engaging community stakeholders

In this section, we present eight lessons learned, with supporting evaluation data, from our experience conducting the SD GMB workshops with community stakeholders. Table 3 provides a summary of lessons learned and supporting evaluation results.

**Table 3:** Summary of the key lessons learned supporting evaluation data.

Lesson	Description	Supporting evaluation data
Center community needs to strengthen engagement and facilitation	Meaningful engagement requires intentional, community-centered design that prioritizes respect, comfort, and inclusion	Evaluation survey results indicate that most stakeholders were satisfied with their participation and found the facilitation approach inclusive and effective
Equip stakeholders early to enhance participation and confidence	Stakeholders need clear preparation to engage meaningfully in complex modeling activities	Survey responses suggest that orientation activities helped stakeholders feel more prepared and confident entering the workshops
Remove participation barriers through meaningful support	Providing compensation and logistical support reduces barriers and fosters inclusion	Stakeholders reported that compensation and other supports, such as childcare, meals, and stipends, were sufficient
Foster co-learning to build capacity and empower action	Stakeholders should gain tools and confidence to apply systems thinking in their own work	Evaluation data show that most stakeholders intended to apply what they learned, reflecting increased capacity and empowerment
Create safe spaces to support emotional well-being and trust	Trauma-informed facilitation and structured reflection foster psychological safety and transparency	Open-ended responses on evaluation surveys highlight appreciation for structured breaks and mindfulness practices that supported emotional well-being
Expand definitions of expertise to enrich systems thinking	Including non-traditional voices broadens understanding and strengthens model relevance	Stakeholders valued the heterogeneity of stakeholders and felt that all perspectives were respected and included
Build Iterative processes for sustained collaboration	Iterative engagement ensures stakeholder insights remain central to model development	Stakeholders emphasized the importance of ongoing engagement and noted that their input shaped the evolving model
Support transformative shifts in stakeholder understanding	Time and reflection enable stakeholders to move from skepticism to agency and structural awareness	Open-ended responses across multiple evaluation surveys illustrated a shift in understanding, with many expressing increased trust and recognition of systemic issues

### Center community needs to strengthen engagement and facilitation

One foundational lesson is that meaningful stakeholder engagement goes beyond technical planning; it requires intentional, community-centered design. From the outset, our team prioritized stakeholder respect, comfort, and inclusion during workshops. Data from the final evaluation survey suggest this approach resonated: 96% of stakeholders (26 out of 27) reported being satisfied with their involvement, 100% (27 out of 27) reported the experience was a good use of their time, and 96% (26 out of 27) expressed interest in participating in future phases. These results point to the effectiveness of our facilitation approach, which emphasized listening, inclusivity, and shared purpose. The results from the facilitation quality subscale on the final evaluation survey (*n* = 27; M = 1.30; SD = 0.375) suggest that our team was not only technically competent but also deeply engaged, relationally skilled, context-sensitive, and committed to co-production and empowerment. The mean facilitation quality score did not differ between individuals with lived experience (M = 1.30; SD = 0.377) and those without lived experience (M = 1.30; SD = 0.385). This profile aligns with best practices in community-centered facilitation and is likely to foster sustained participation, localized innovation, and trust in the improvement process.

### Equip stakeholders early to enhance participation and confidence

Another key lesson was that stakeholders need clear preparation to engage meaningfully in complex modeling activities. In these two projects, we developed visual orientation materials, facilitated pre-workshop calls, and provided user-friendly binders with systems thinking tools. Data from the final evaluation survey indicate that 74% of stakeholders (20 out of 27) reported they attended the orientation call. Of the 20 stakeholders who reported attending orientation calls, 90% (18 out of 20 respondents) reported that the pre-orientation call helped prepare them for the workshops. This experience underscores the importance of accessible, well-planned onboarding to promote confident and informed participation.

### Remove participation barriers through meaningful support

Another lesson is the importance of providing stakeholders with adequate compensation and support. While there is no universal standard for what constitutes “adequate,” it is essential to gauge this directly from the stakeholders involved. Transparent reporting in this area remains limited, and our work contributes to filling that gap. Data from the final evaluation survey indicated that 100% of stakeholders (*n* = 27) felt the compensation and additional supports—such as childcare, transportation, lactation spaces, and meals—were sufficient. This suggests that when teams attend to logistical and financial barriers, it communicates respect for stakeholders' time and enables more inclusive participation.

### Foster co-learning to build capacity and empower action

Stakeholders should leave with more than a sense of contribution—they should gain knowledge, tools, or inspiration they can apply in their own work. This principle of co-learning was a priority in our design. Data from the final evaluation survey showed that 88% of stakeholders (23 out of 26) intended to apply what they learned to their own work. Notably, 75% of stakeholders with lived experience (6 out of 8) and 94% of stakeholders without lived experience (17 out of 18) reported intentions to apply what they learned. The average score on the capacity building scale on the final evaluation survey was 4.20 (*n* = 27; SD = 0.635), suggesting that the workshop effectively fostered capacity building by enhancing stakeholders’ knowledge, strengthening collaborative relationships, and increasing their confidence and ability to contribute meaningfully to patient care and team-level impact. The average capacity building score was similar among stakeholders with lived experience (M = 4.3; SD = 0.667) and stakeholders with no lived experience (M = 4.0; SD = 0.620). This measure captures both individual growth and collective efficacy—key indicators of successful capacity building in health improvement contexts. We even had some stakeholders express interest in downloading the modeling software that was used by the team for facilitation (i.e., Vensim) and learning how to use it within their organizations.

### Create safe spaces to support emotional well-being and trust

Because the workshops addressed emotionally charged topics, including traumatic lived experiences, it was essential to create a psychologically safe space. We incorporated mindfulness exercises, offered a meditation room, and designed structured breaks to support stakeholders' well-being. We wanted to recognize that this is not a trivial issue but rather a psychologically heavy topic especially for people with a lived experience. As one stakeholder mentioned in the final evaluation survey, “I appreciate the breaks since it was a lot of heavy discussion.” This suggests that trauma-informed facilitation is not ancillary—it is core to ethical and effective engagement. Structured reflection activities—such as the *Concerns and Hopes* exercise—helped surface expectations, confront issues of institutional trustworthiness, and guide the process. This suggests that intentional facilitation strategies can help build transparency and shared ownership, especially when working with diverse or previously siloed stakeholders.

### Expand definitions of expertise to enrich systems thinking

We also learned the value of expanding our definition of expertise. Our recruitment strategy intentionally engaged what we termed “unusual suspects”—individuals with lived experience or expertise in non-medical domains (e.g., housing). One stakeholder reported in the final evaluation survey, “diversity of stakeholders was good, and I felt that everyone's opinion was valued according to experience.” This approach broadened the lens through which we understood maternal health outcomes and helped ensure the model was rooted in intersectional realities.

### Build iterative processes for sustained collaboration

Our process was designed as iterative and ongoing, not a one-off engagement. We conducted multiple modeling sessions and communicated a clear intention to continue engaging stakeholders in refinement and implementation. The iterative nature of the process ensured that stakeholder insights remained central to model development and validation. This reinforces the lesson that participatory processes must be built for continuity and long-term impact.

### Support transformative shifts in stakeholder understanding

Finally, one of the most powerful findings was the transformation in stakeholder perspectives over time. The two-session structure (2.5 days) allowed stakeholders to move from confusion and skepticism to clarity and collective action. At the end of the first day of workshop Session 1, many grappled with the complexity of systems modeling. One stakeholder shared the challenge of “coalescing into a sort of coherent model,” while another voiced concern about “remembering the techniques and meanings around the model structure.” Others questioned whether the model would have any real-world relevance, stating, “[My concern is that] it will not be effective past these interactions.” By the end of the second day of workshop Session 1, the conversation had shifted from technical difficulties to system-level challenges. Stakeholders began acknowledging the broader institutional and policy-related barriers to change. “[My concern is that] there are several systems in place that work against the common goal,” noted a stakeholder, while another shared, “Texas is not exactly forthcoming with data.” The modeling method was increasingly seen as viable, and stakeholders' trust in the process appeared to grow.

By the end of the second workshop session, transformation was evident. Stakeholders expressed hope, agency, and belief in the collective. One stakeholder stated, “I believe an impactful group of community members have been assembled to drive results that will lower SMM rates.” There was also a deeper confrontation of structural forces: “I am happy we got a chance to dive into the roots of racism and it is still affecting us, Black communities, today. It was interesting seeing the differences in the small groups’ causal models.” Another stakeholder reported, “What resonated with me the most was just how much racism plays a role in SMM and how deeply American society and systems are embedded in racism.” Others emphasized continued inclusion, especially of mothers with lived experience. This progression—from uncertainty to empowerment—highlights the value of giving communities time to build shared understanding, deepen relationships, and shape a vision for change.

## Discussion

This study demonstrates how system dynamics modeling, grounded in CBPR principles, can be used to engage diverse stakeholders—particularly Black women—in addressing adverse maternal health outcomes. Our findings contribute to the growing literature on community-engaged system dynamics group model building in the field of maternal and child health.

By embedding CBPR principles throughout the workshop design and facilitation process, we fostered equitable partnerships, mutual learning, and shared ownership of knowledge (33). These principles were operationalized through inclusive recruitment, wraparound supports, and trauma-informed facilitation strategies. The result was a process that not only generated systems insights but also honored community expertise and built relational trust (34). Participant selection was enhanced by including a “community consultant” on the research team, which confirms best practices reported elsewhere (41). The workshops also created conditions for transformative learning. Drawing on transformative learning theory, we observed how structured reflection, dialogue, and collaborative modeling enabled stakeholders to critically examine assumptions, recognize systemic inequities, and develop a stronger sense of agency (22, 42). Stakeholders moved from initial skepticism to a deeper understanding of structural drivers and a collective vision for change—hallmarks of transformative engagement.

Our approach also offers a replicable model for trauma-informed, person-centered facilitation. By integrating mindfulness, structured reflection, and logistical supports, we created psychologically safe spaces that honored the lived experiences of stakeholders. This design may be particularly valuable for other teams working in emotionally charged or historically marginalized contexts. A key implication of this work is the potential for SD GMB workshop outputs—such as CLDs, BOTGs, and model boundary charts (MBCs)—to inform the development of simulation dashboards. These tools can support decision-makers in testing policy scenarios and identifying high-leverage interventions to reduce maternal health disparities.

### Limitations

Several limitations warrant consideration. First, despite efforts to recruit a diverse group of stakeholders, key institutional actors—such as local policymakers—were less involved than desired due to scheduling conflicts. Their absence may limit the scalability and policy relevance of the models. Future work should explore alternative engagement strategies (e.g., asynchronous input or policy-focused convenings) to incorporate these critical perspectives. Second, while guided by CBPR principles, participatory system dynamics modeling involves inherent power dynamics that can shape whose voices influence the model. Even well-intentioned facilitation may unintentionally reflect technical biases. Future efforts should explore ways to decentralize facilitation and share modeling authority with community stakeholders across all phases, including diagraming, interpretation, and revision. Third, this study does not assess whether the models will lead to policy or practice change. Participatory modeling is often valued for its process, but future research should examine downstream impacts on intervention design, decision-making, and implementation. Our evaluation relied on immediate post-workshop surveys capturing self-reported knowledge, satisfaction, and intent to apply learning. While informative, these data do not reflect long-term changes. Longitudinal, mixed-methods approaches are needed to assess sustained impact, including whether stakeholders apply systems thinking, influence institutional change, or contribute to policy outcomes. Finally, although we observed shifts in stakeholder perspectives—from skepticism to agency—the study lacked a guiding theoretical framework to interpret this change. Theories of transformative learning (22) could help explain how and why stakeholder engagement evolved. Future studies should integrate such frameworks to better understand the mechanisms of change.

### Future directions

Building on this work, we are considering sharing our facilitation model and related materials through an open-access repository [e.g., Zimmerman et al. (26)]. This would allow other researchers and practitioners to access annotated agendas, recruitment templates, and community-centered strategies. In parallel, we are working to quantify the system dynamics models and plan to convene a series of learning labs with stakeholders to explore intervention data and test policy scenarios. We plan to expand our stakeholder base to include additional institutional decision-makers and policymakers. These efforts will converge in the design and implementation of an interactive dashboard that enables users to visualize feedback loops, simulate long-term outcomes, and identify high-leverage strategies to improve maternal health outcomes.

### Conclusion

This paper advances efforts to make systems thinking and system dynamics more accessible to non-technical audiences. It does so by actively involving community stakeholders in the modeling process and offering a practical example of participatory systems thinking in action (9, 17–20, 25).

The projects featured in this paper integrated community-based participatory research with system dynamics modeling while centering Black women's lived experiences. By design, we fostered transformative learning and co-production of systems insights. As we move toward quantifying models and developing interactive policy dashboards, this work lays the foundation for sustainable, community-driven approaches to maternal health research, policy, and practice.

## Data Availability

The raw data supporting the conclusions of this article will be made available by the authors, without undue reservation.

## Footnotes

1 Stakeholders participating as representatives of government agencies declined compensation.

## References

[1] Gunja MZ Gumas ED Masitha R Zephyrin LC. Insights into the U.S. Maternal Mortality Crisis: An International Comparison. Washington, D.C.: The Commonwealth Fund (2024). Available online at: https://www.commonwealthfund.org/publications/issue-briefs/2024/jun/insights-us-maternal-mortality-crisis-international-comparison (Accessed June 19, 2025).

[2] Admon LK Winkelman TNA Zivin K Terplan M Mhyre JM Dalton VK. Racial and ethnic disparities in the incidence of severe maternal morbidity in the United States, 2012–2015. Obstet Gynecol. (2018) 5:1158–66. 10.1097/AOG.0000000000002937 30303912

[3] Briller JE. Severe maternal cardiovascular morbidity. JACC Adv. (2022) 1(4):100124. 10.1016/j.jacadv.2022.100124 38939707 PMC11198691

[4] Malhamé I Mehta N Raker CA Hardy EJ Spalding H Bouvier BA Identifying cardiovascular severe maternal morbidity in epidemiologic studies. Paediatr Perinat Epidemiol. (2020) 34(4):452–9. 10.1111/ppe.12571 31971615

[5] Alio AP Richman AR Clayton HB Jeffers DF Wathington DJ Salihu HM. An ecological approach to understanding black–white disparities in perinatal mortality. Matern Child Health J. (2010) 14(4):557–66. 10.1007/s10995-009-0495-9 19562474

[6] Bailey ZD Krieger N Agénor M Graves J Linos N Bassett MT. Structural racism and health inequities in the USA: evidence and interventions. Lancet. (2017) 389(10077):1453–63. 10.1016/S0140-6736(17)30569-X 28402827

[7] Crear-Perry J Correa-de-Araujo R Lewis Johnson T McLemore MR Neilson E Wallace M. Social and structural determinants of health inequities in maternal health. J Womens Health. (2021) 30(2):230–5. 10.1089/jwh.2020.8882 PMC8020519 33181043

[8] Davis DA. Obstetric racism: the racial politics of pregnancy, labor, and birthing. Med Anthropol. (2019) 38(7):560–73. 10.1080/01459740.2018.1549389 30521376

[9] Lemke MK Brown KK Fallah-Fini S Hall A Obasanya M. Complex systems and participatory approaches to address maternal health disparities: findings from a system dynamics group model building project in North Texas. Am J Community Psychol. (2023) 71(3–4):303–16. 10.1002/ajcp.12636 36378746

[10] Lu MC Kotelchuck M Hogan V Jones L Wright K Halfon N. Closing the black-white gap in birth outcomes: a life-course approach. Ethn Dis. (2010) 20(1 Suppl 2):S2-62–76. PMID . 20629248 PMC4443479

[11] Clark EC Cranston E Polin T Ndumbe-Eyoh S MacDonald D Betker C Structural interventions that affect racial inequities and their impact on population health outcomes: a systematic review. BMC Public Health. (2022) 22(1):2162. 10.1186/s12889-022-14603-w 36424559 PMC9685079

[12] Gordon RD Hatab J Voisin CE Gillespie SL Bunger A Rodriguez Miranda M Postpartum primary care in the United States: a scoping review of the evidence base and opportunities. J Womens Health. (2025). 10.1089/jwh.2024.0813 40421711

[13] Yalley AA Jarašiūnaitė-Fedosejeva G Kömürcü-Akik B de Abreu L. Addressing obstetric violence: a scoping review of interventions in healthcare and their impact on maternal care quality. Front Public Health. (2024) 12. 10.3389/fpubh.2024.1388858 38979044 PMC11228167

[14] Apostolopoulos Y Lemke MK Hosseinichimeh N Harvey IS Lich KH Brown J. Embracing causal complexity in health disparities: metabolic syndemics and structural prevention in rural minority communities. Prev Sci. (2018) 19(8):1019–29. 10.1007/s11121-018-0924-3 29959717

[15] Lofgren E. Systems dynamics models. In: El-Sayed AM Galea S, editors. Systems Science and Population Health. Oxford: Oxford University Press (2017). p. 77–86. 10.1093/acprof:oso/9780190492397.003.0007

[16] Sooka C Rwashana-Semwanga A. Modeling the dynamics of maternal healthcare in Uganda: a system dynamics approach. World J Model Simul. (2011) 7(3):163–72.

[17] Brown KK Lemke MK Fallah-Fini S Hall A Obasanya M. Planning, implementing, and evaluating an online group-model-building workshop during the COVID-19 pandemic: celebrating successes and learning from shortcomings. Syst Dyn Rev. (2022) 38(1):93. 10.1002/sdr.1704 35599641 PMC9111080

[18] Hyder A Smith M Sealy-Jefferson S Hood RB Chettri S Dundon A Community-based systems dynamics for reproductive health: an example from urban Ohio. Prog Community Health Partnersh. (2022) 16(3):361–83. 10.1353/cpr.2022.0053 36120879 PMC9709633

[19] Kroelinger CD Rankin KM Chambers DA Diez Roux AV Hughes K Grigorescu V. Using the principles of complex systems thinking and implementation science to enhance maternal and child health program planning and delivery. Matern Child Health J. (2014) 18(7):1560–4. 10.1007/s10995-014-1586-9 25108501 PMC4295498

[20] Hassmiller Lich K Urban JB Frerichs L Dave G. Extending systems thinking in planning and evaluation using group concept mapping and system dynamics to tackle complex problems. Eval Program Plann. (2017) 60:254–64. 10.1016/j.evalprogplan.2016.10.008 27825622

[21] Estrada-Magbanua WM Huang TTK Lounsbury DW Zito P Iftikhar P El-Bassel N Application of group model building in implementation research: a systematic review of the public health and healthcare literature. PLoS One. (2023) 18(8):e0284765. 10.1371/journal.pone.0284765 37590193 PMC10434911

[22] van Bruggen A Nikolic I Kwakkel J. Modeling with stakeholders for transformative change. Sustainability. (2019) 11(3):825. 10.3390/su11030825

[23] Billups S Thelamour B Thibodeau P Durgin FH. On intersectionality: visualizing the invisibility of black women. Cogn Res Princ Implic. (2022) 7(1):100. 10.1186/s41235-022-00450-1 36435861 PMC9701302

[24] Israel BA Schulz AJ Parker EA Becker AB. “Critical issues in developing and following community-based participatory research principles”. In: Minkler M Wallerstein N, editors. Community-Based Participatory Research for Health. San Francisco, CA: Jossey-Bass (2008). p. 47–62. Available online at: https://www.scholars.northwestern.edu/en/publications/critical-issues-in-developing-and-following-community-based-parti-2 (Accessed Jan. 3, 2020).

[25] Zimmerman L Lounsbury DW Rosen CS Kimerling R Trafton JA Lindley SE. Participatory system dynamics modeling: increasing stakeholder engagement and precision to improve implementation planning in systems. Adm Policy Ment Health. (2016) 43(6):834–49. 10.1007/s10488-016-0754-1 27480546 PMC8344379

[26] Zimmerman L Lounsbury D. Team Participatory System Dynamics Repository. GitHub (2018). Available online at: https://github.com/lzim/teampsd/blob/master/mtl_facilitate_workgroup/evaluation/measures.md (Accessed February 15, 2025).

[27] Collins SR Radley DC Roy S Zephrin LC Shah A. 2024 State Scorecard on Women’s Health and Reproductive Care. New York, NY: Commonwealth Fund (2024). Available online at: https://www.commonwealthfund.org/publications/scorecard/2024/jul/2024-state-scorecard-womens-health-and-reproductive-care (Accessed July 27, 2025).

[28] Texas Department of State Health Services. Regional Analysis of Maternal and Infant Health in Texas, Public Health Region 2/3. Austin, TX: Texas Department of State Health Services (2018). Available online at: https://www.dshs.texas.gov/mch/epi/docs/02-Regional-Analysis-of-Maternal-and-Infant-Health-in-Texas_PHR-2-3.pdf (Accessed June 11, 2021).

[29] Census Reporter. Census Reporter Profile on Dallas-Fort Worth-Arlington Metro Area. Census Reporter (2019). Available online at: http://censusreporter.org/profiles/31000US19100-dallas-fort-worth-arlington-tx-metro-area/ (Accessed May 13, 2021).

[30] Texas Department of State Health Services, Maternal Mortality and Morbidity Review Committee. Texas Maternal Mortality and Morbidity Review Committee and Department of State Health Services Joint Biennial Report. Austin, TX: Texas Department of State Health Services (2020). Available online at: https://www.dshs.texas.gov/legislative/2020-Reports/DSHS-MMMRC-2020.pdf (Accessed June 10, 2021).

[31] Bryson JM. What to do when stakeholders matter: stakeholder identification and analysis techniques. Public Manag Rev. (2004) 6(1):21–53. 10.1080/14719030410001675722

[32] Homer J. Best practices in system dynamics modeling, revisited: a practitioner’s view. Syst Dyn Rev. (2019) 35(2):177–81. 10.1002/sdr.1630

[33] Hovmand PS. Community Based System Dynamics. New York, NY: Springer (2014).

[34] Weeks MR Li J Lounsbury D Green HD Abbott M Berman M Using participatory system dynamics modeling to examine the local HIV test and treatment care continuum in order to reduce community viral load. Am J Community Psychol. (2017) 60(3–4):584–98. 10.1002/ajcp.12204 29154393 PMC5729085

[35] Washington University in St. Louis. Changing Systems. (2016). Available online at: https://www.youtube.com/watch?v=*S*-EC-rtqsrc (Accessed July 27, 2025).

[36] Intuit. Mailchimp. Atlanta, GA (2024). Available online at: https://www.mailchimp.com

[37] Thinking Tools Studio. Habits of a Systems Thinker. Tucson, AZ: Thinking Tools Studio (n.d.). Available online at: https://thinkingtoolsstudio.org/ (Accessed February 15, 2025).

[38] Scriptapedia. Scriptapedia—Wikibooks, open books for an open world. (2022). Available online at: https://en.wikibooks.org/wiki/Scriptapedia (Accessed July 27, 2025).

[39] United States Agency for International Development. The 5Rs Framework in the Program Cycle. Washington, DC: USAID Bureau for Policy Planning and Learning (2016).

[40] QuestionPro. QuestionPro Survey Software. Austin, TX (2024). Available online at: https://www.questionpro.com/

[41] Andersen DF Richardson GP. Scripts for group model building. Syst Dyn Rev. (1997) 13(2):107–29. 10.1002/(SICI)1099-1727(199722)13:2<107::AID-SDR120>3.0.CO;2-7

[42] Mezirow J. Transformative learning: theory to practice. New Dir Adult Contin Educ. (1997) 1997(74):5–12. 10.1002/ace.7401

[43] Lemke MK Brown KK Fallah-Fini S Lounsbury DW Kindratt T Lambert T Understanding and addressing severe mMaternal mMorbidity among Black women in Texas: findings from a system dynamics group model building study. J Racial and Ethnic Health Disparities. (2025). 10.1007/s40615-025-02568-6 PMC13245379 40731190

