# Supplementary material for: Engaging communities, modeling systems: lessons from system dynamics modeling on maternal health in Texas
Source: Front Glob Womens Health. 2025 Sep 25;6:1577568. doi: 10.3389/fgwh.2025.1577568 (PMC12507813; doi:10.3389/fgwh.2025.1577568)
Supplement: Supplementary file 1 [file Datasheet1.pdf]

PROJECT IMPACT: CARDIOVASCULAR SEVERE MATERNAL MORBIDITYPost-Session Feedback FormInstructions: As you rate today's session, please respond to each item. We will use your ratings and suggestions to make improvements. Thank you!

Please enter your assigned Participant ID number.Note: It is located in your workshop binder.

Please select the session that you attended.

1. Day 1: March 8
2. Day 2: March 9
3. I did not attend either day

Please respond to each question about today's session.

	Very Good	Good	Fair	Poor	Very Poor	N/A
The overall quality of today’s session was...	❑	❑	❑	❑	❑	❑
In general, the level of group member participation was...	❑	❑	❑	❑	❑	❑
The degree to which group members’ ideas were understood and acknowledged during today’s session was...	❑	❑	❑	❑	❑	❑
Responsiveness to questions group members asked today was...	❑	❑	❑	❑	❑	❑
You felt the quality of your participation in today’s session was...	❑	❑	❑	❑	❑	❑
At the present time, your understanding of the model building process is..	❑	❑	❑	❑	❑	❑
At the present time, your understanding of the components of system dynamics models is..	❑	❑	❑	❑	❑	❑
The potential for the group to develop a useful system dynamics model about cardiovascular severe maternal morbidity among Black women is...	❑	❑	❑	❑	❑	❑
The potential for the group to identify the necessary data to simulate the model is__	❑	❑	❑	❑	❑	❑
The potential of modeling to improve decision-making (as compared to decision-making without modeling is)	❑	❑	❑	❑	❑	❑
The potential of modeling to save time and effort by running model simulations before we make changes (as compared to implementing changes without modeling) is:	❑	❑	❑	❑	❑	❑

After today's session, my biggest concern about the modeling process is...

After today's session, I believe the most useful aspect of the modeling process is likely to be...

Do you have any suggestions for improvements to the session, process, facilitation or other aspects of today?

Please share any personal reflections or insights from this session. For instance, what stuck with you today and why? This question helps us to understand how your thoughts and reflections may evolve over time.

Project IMPACT: CARDIOVASCULAR SEVERE MATERNAL MORBIDITYRegistration and Baseline Evaluation SurveyThank you for agreeing to participate in this research project! Please take 10-15 minutes to complete this registration form, which also includes baseline evaluation questions. Please let us know if you have any questions or concerns, projectimpact@uta.edu.

REGISTRATION QUESTIONS

Please enter your full name (and any credentials that should be listed behind your name)

Please provide your preferred pronouns.

Which of the following BEST describes your gender identity?

1. Prefer not to answer
2. Woman
3. Man
4. Non-Binary or Gender Non-Conforming
5. Gender Neutral
6. Gender Queer
7. Not Listed __________

Are you a person of Trans experience?

1. Prefer not to answer
2. Yes
3. No

Do you have Hispanic or Spanish origins/ancestry?

1. Prefer not to answer
2. Yes
3. No

Please select the option that best characters your "race".Note: We recognize that race is a social construct. Also, this information will be used to ensure transparency in project participation by reporting the racial demographic of participating experts.

1. Prefer not to answer
2. American Indian or Alaska Native or Indigenous
3. East Asian

The University of Texas at Arlington - Legal and Privacy Notice

4. South Asian
5. Black or African American
6. Latino/a/e/x
7. Native Hawaiian or Pacific Islander
8. Middle Eastern or North African
9. White
10. Multi-Racial

Please select your age group.

1. Prefer not to answer
2. 18 - 29 years old
3. 30 - 39 years old
4. 40 - 49 years old
5. 50 - 59 years old
6. 60 - 69 years old
7. 70 - 79 years old
8. 80 or older

Accommodations

Do you need any Americans with Disabilities (ACT) accommodations to successfully participate in this project?

1. Yes
2. No

Please tell us what ADA accommodations you will need to successfully participate in this project.

Will you need onsite childcare in order to participate in this project?

1. Yes
2. No

Will you need local transportation or transportation support to participate in this project?

1. Yes
2. No

Will you need a lactation (breast/chest feeding) room?

1. Yes
2. No

Which of the following days will you NOT be able to attend?

1. Friday, March 8th
2. Saturday, March 9th
3. Friday, March 22nd

Dietary Restrictions/Food AllergiesIf applicable, please describe any dietary restrictions (e.g., nut allergy, no pork, gluten-free). Please provide a specific response because this information will be used to inform catering orders.

Will you need to park a vehicle at the meeting site?We are requesting this information because if you do participate, our team will need to arrange parking permits so that your vehicle is not towed or fined. If you do not end up participating, this information will be permanently deleted.

1. Yes
2. No

Please list the full name of the person on the vehicle registration.

Please provide the vehicle MAKE/BRAND (e.g., Honda, Ford, Toyota)

Please provide the vehicle MODEL (e.g., CRV, Camry, Equinox)

Please provide the vehicle LICENSE PLATE NUMBER AND STATE (e.g., ABC 5890 Texas).

Which of the following best describes your participation?

1. I am participating as a community member who has personal/lived experience.
2. I am participating as a representative of a profession, business, agency, or organization.

Please list the full name of the business, agency, organization, or institution (or profession) that you are representing.

Please list your profession or job title.

At what geographic scale do you/your organization work?

1. City or County
2. State (including across regions within the state)
3. National
4. International

Is your business, agency, organization, or institution woman-led or woman-owned?

1. Yes
2. No

Is your business, agency, organization, or institution Black-led or Black-owned?

1. Yes
2. No

Is your business, agency, organization, or institution person of color-led or person of color-owned?

1. Yes
2. No

We understand that your business, agency, or organization may work across multiple sectors. Please select the option that BEST

describes the sector in which your business, agency, or organization operates?   Limit to 2 categories.

1. Advocacy or Lobbying
2. Arts, Entertainment, and Recreation (e.g., museums,   etc.)
3. Business (including small business, private business, business/economic development)
4. Education/Academia
5. Government or Public Sector (e.g., law enforcement, public office)
6. Faith or Religious Institution
7. Health Care (including mental health care, behavioral health care)
8. Information Services (e.g., libraries, historical societies/historians)
9. Law
10. Public Health (including governmental public health and environmental health)
11. Social Services (e.g., child protective services,)
12. Technology (including Health Technology, Informatics)
13. Transportation or Infrastructure (e.g., housing)
14. Non-profit sector
15. Other __________
16. N/A

Who made the final decision for you to participate in this project?

1. I made the final decision
2. My organization's senior leadership
3. My direct division/program supervisor
4. Other __________
5. N/A

Have you ever participate in a project that involves "group model building" or "system dynamics modeling" ?

1. Yes
2. No

BASELINE ASSESSMENT

Please answer the following questions to the best of your ability. If you do not know the correct answer, you can select "Don't Know".

_________ refers to a way of understanding how societal problems reflect a system that is made up of constantly changing, interrelated factors.

1. System Dynamics
2. Systems Thinking
3. Complex Perspectives
4. Don't Know

____ is a type of computer-aided approach for policy design and analysis intended to help people make better decisions when confronted with complex, dynamic systems.

1. System Dynamics
2. Complex Systems Science
3. Group Modeling
4. Don't Know

The University of Texas at Arlington - Legal and Privacy Notice

______ is a participatory method aimed at engaging community stakeholders to collectively identify the causes of complex problems.

1. Participatory Group Model Building
2. System Modeling
3. Participatory Research
4. Don't Know

_______ is a diagram that aids in visualizing how different variables interact to cause a system behavior or outcome.

1. System Loop Diagram
2. Causal Loop Diagram
3. Feedback Loop Diagram
4. Don't Know

_____ refers to the totality of ways in which societies foster racial discrimination through mutually-reinforcing systems of housing, education, employment, earnings, credit, health care, and criminal justice.

1. Systems
2. Structural Racism
3. Oppression
4. Don't Know

The tendency of systems to defeat the well-intended policies and programs that have been designed to improve them is known as _______.

1. System Failure
2. Implementation Failure
3. Policy Resistance
4. Don't Know

Please read each of the statements and answer each one as truthfully as possible.

When I want to make an improvement, I seek everyone's view of the situation

1. Never
2. Seldom
3. Some of the time
4. Often
5. Most of the time

When I want to make an improvement, I look beyond a specific event to determine the cause of the problem.

1. Never
2. Seldom
3. Some of the time
4. Often
5. Most of the time

When I want to make an improvement, I think understanding how the chain of events occur is important.

1. Never
2. Seldom

3. Some of the time
4. Often
5. Most of the time

When I want to make an improvement, I include other people to find a solution.

1. Never
2. Seldom
3. Some of the time
4. Often
5. Most of the time

When I want to make an improvement, I think recurring patterns are more important than any one specific event.

1. Never
2. Seldom
3. Some of the time
4. Often
5. Most of the time

When I want to make an improvement, I think of the problem at hand as a series of connected issues.

1. Never
2. Seldom
3. Some of the time
4. Often
5. Most of the time

When I want to make an improvement, I consider the cause and effect that is occurring in a situation.

1. Never
2. Seldom
3. Some of the time
4. Often
5. Most of the time

When I want to make an improvement, I consider the relationships among the people in and outside of my organization.

1. Never
2. Seldom
3. Some of the time
4. Often
5. Most of the time

When I want to make an improvement, I think that systems are constantly changing.

1. Never
2. Seldom
3. Some of the time
4. Often
5. Most of the time

When I want to make an improvement, I propose solutions that affect the environment, not specific individuals.

1. Never
2. Seldom
3. Some of the time
4. Often
5. Most of the time

When I want to make an improvement, I keep in mind that proposed changes can affect the whole system.

1. Never
2. Seldom
3. Some of the time
4. Often
5. Most of the time

When I want to make an improvement, I think more than one or two people are needed to have success.

1. Never
2. Seldom
3. Some of the time
4. Often
5. Most of the time

When I want to make an improvement, I keep the mission and purpose of the organization in mind.

1. Never
2. Seldom
3. Some of the time
4. Often
5. Most of the time

When I want to make an improvement, I think small changes can produce important results.

1. Never
2. Seldom
3. Some of the time
4. Often
5. Most of the time

When I want to make an improvement, I consider how multiple changes affect each other.

1. Never
2. Seldom
3. Some of the time
4. Often
5. Most of the time

When I want to make an improvement, I think about how different people might be affected by the improvement.

1. Never
2. Seldom
3. Some of the time
4. Often
5. Most of the time

When I want to make an improvement, I try strategies that do not rely on people's memory.

1. Never
2. Seldom
3. Some of the time
4. Often
5. Most of the time

When I want to make an improvement, I recognize system problems are influenced by past events.

1. Never
2. Seldom
3. Some of the time
4. Often
5. Most of the time

When I want to make an improvement, I consider the past history and culture of the environment.

1. Never
2. Seldom
3. Some of the time
4. Often
5. Most of the time

When I want to make an improvement, I consider that the same action can have different effects over time, depending on the state of the system.

1. Never
2. Seldom
3. Some of the time
4. Often
5. Most of the time

PROJECT IMPACT: CARDIOVASCULAR SEVERE MATERNAL MORBIDITY
Final Evaluation SurveyThank you for participating in the System Dynamics Group Model Building Workshops, which are the first phase of Project IMPACT. Your completion of this survey will provide us with valuable information about what we are doing well and what improvements we can make going forward.This final evaluation survey will ask you questions about the final workshop session on March 22nd (if you attended it) and questions about your perceptions about the overall project experience. Please be prepared to enter your ID number. This final evaluation survey will take about 15 minutes to complete. If you encounter any technical issues with this survey, please contact us at projectimpact@uta.edu.Thank you,The Project IMPACT teamprojectimpact@uta.edu

Please enter your assigned Participant ID number.Note: It is located in your workshop binder and in your confirmation email.

Did you attend the final workshop session on March 22nd?

1. Yes
2. No

Session Evaluation

Please respond to each question about today's session.

	Very Good	Good	Fair	Poor	Very Poor	N/A
The overall quality of today’s session was...	❑	❑	❑	❑	❑	❑
In general, the level of group member participation was...	❑	❑	❑	❑	❑	❑
The degree to which group members’ ideas were understood and acknowledged during today’s session was...	❑	❑	❑	❑	❑	❑
Responsiveness to questions group members asked today was...	❑	❑	❑	❑	❑	❑
You felt the quality of your participation in today’s session was...	❑	❑	❑	❑	❑	❑
At the present time, your understanding of the model building process is..	❑	❑	❑	❑	❑	❑
At the present time, your understanding of the components of system dynamics models is..	❑	❑	❑	❑	❑	❑
The potential for the group to develop a useful system dynamics model about cardiovascular severe maternal morbidity among Black women is...	❑	❑	❑	❑	❑	❑
The potential for the group to identify the necessary data to simulate the model is__	❑	❑	❑	❑	❑	❑
The potential of modeling to improve decision-making (as compared to decision-making without modeling is)	❑	❑	❑	❑	❑	❑
The potential of modeling to save time and effort by running model simulations before we	❑	❑	❑	❑	❑	❑

make changes (as compared to implementing changes without modeling) is:						

Please share any feedback that you think is important for the team to know.

Please share any personal reflections or insights from this session. For instance, what stuck with you today and why?

Project Evaluation

Did you attend (or view the recording of) the Project IMPACT orientation meeting?

1. Yes
2. No
3. Not Sure

Was the orientation meeting/recording helpful to you?

1. Yes
2. No

How would you rate the compensation and other supports (e.g., meals, parking, childcare, transporation suppot, etc.) offered as a part of this project?

1. Not at all sufficient
2. Somewhat sufficient
3. Sufficient

How satisfied or dissatisfied are you with the quality of this phase of the project?

1. Very dissatisfied
2. dissatisfied
3. Neutral
4. Satisfied
5. Very satisfied

Based on your experience, would you be interested in participating in similar projects in the future?

1. Yes
2. No
3. Not Sure

The objectives of this phase (group model building workshops) were to convene community stakeholders to (1) identify the factors and forces driving CSMM among Black women in Texas [5Rs], (2) explain how these factors and forces (and trends in SMM) have fluctuated over time [Behavior Over Time Graphs], (3) to illustrate how those factors and forces relate to one another over time…perhaps in nonlinear or cyclical ways [Causal Mapping], and (4) identify targets for action and additional sources of evidence or information.In your opinion, were the objectives met for the phase of the project in which you participated?

1. Yes, definitely
2. Yes, somewhat
3. No
4. Not Sure

Do you intend to apply any concepts that you learned during this project to your own work?

1. Yes
2. No
3. Not Sure

This project was a good use of my time.

1. Strongly agree
2. Agree
3. Neither agree nor disagree
4. Disagree
5. Strongly Disagree

In a few months, would you be interested in participating in a group meeting that features community stakeholders from both cohorts (CSMM and SMM) to co-learn and share lessons learned?

1. Yes
2. No
3. Maybe

We want to stay engaged and in communication with you after the end of these workshops. Please let us know the best format to do this.

1. What's App
2. Facebook private group
3. LinkedIn private group
4. Teams space
5. Listserv
6. Newsletter
7. Periodic virtual check-in meetings
8. Platform such as Google Classrooms or Moodle
9. Other __________

FacilitationBased on your overall experience with the project, please rate the following statements about the facilitation and core partnership values.

	Strongly Agree	Agree	Neither Agree nor Disagree	Disagree	Strongly Disagree	N/A
Facilitators were clear about the purpose or goal of this project.	❑	❑	❑	❑	❑	❑
Facilitators provided orientation to new members as they joined the sessions.	❑	❑	❑	❑	❑	❑
Facilitators were knowledgeable about our local conditions, values, trends, and prior efforts.	❑	❑	❑	❑	❑	❑
Facilitators established relationships, built trust, and created processes for action.	❑	❑	❑	❑	❑	❑
Facilitators recognized that it is our responsibility and right to act locally to improve conditions.	❑	❑	❑	❑	❑	❑
Facilitators recognized the need to partner with us to create change and improvement.	❑	❑	❑	❑	❑	❑
Facilitators recognized and respected diverse capacities to best use our local resources and make local decisions.	❑	❑	❑	❑	❑	❑

Based on your overall experience with the project, please rate the following statements about the quality group participation, cooperation, and synergy.

	Strongly Agree	Agree	Neither Agree nor Disagree	Disagree	Strongly Disagree
During this project, we showed positive attitudes towards one another.	❑	❑	❑	❑	❑
During this project, everyone participated in our meetings.	❑	❑	❑	❑	❑
During this project, we listened to one another.	❑	❑	❑	❑	❑
During this project, disagreements that occurred during our meetings were constructive.	❑	❑	❑	❑	❑
During this project, when disagreements occurred, we worked together to resolve them.	❑	❑	❑	❑	❑
During this project, even though we did not have total agreement, we did reach a kind of consensus that we all accepted.	❑	❑	❑	❑	❑

Capacity Building

	Not at All	Very Little	Somewhat	To a Large Extent	To a Very Great Extent
I acquired useful knowledge from participation.	❑	❑	❑	❑	❑
I developed valuable relationships from participation.	❑	❑	❑	❑	❑
Participation enhanced my ability to meet the needs of my patients/clients/people I serve.	❑	❑	❑	❑	❑
Participation increased my ability to have a greater impact than I could have on my own.	❑	❑	❑	❑	❑
Participation increased my ability to make a contribution to my organization/team.	❑	❑	❑	❑	❑

When I want to make an improvement:

	Never	Seldom	Some of the time	Often	Most of the time
I seek everyone's view of the situation	❑	❑	❑	❑	❑
I look beyond a specific event to determine the cause of the problem.	❑	❑	❑	❑	❑
I think understanding how the chain of events occur is important.	❑	❑	❑	❑	❑
I include other people to find a solution.	❑	❑	❑	❑	❑
I think recurring patterns are more important than any one specific event.	❑	❑	❑	❑	❑
I think of the problem at hand as a series of connected issues.	❑	❑	❑	❑	❑
I consider the cause and effect that is occurring in a situation.	❑	❑	❑	❑	❑
I consider the relationships among the people in and outside of my organization.	❑	❑	❑	❑	❑
I think that systems are constantly changing.	❑	❑	❑	❑	❑
I propose solutions that affect the environment, not specific individuals.	❑	❑	❑	❑	❑

When I want to make an improvement:

	Never	Seldom	Some of the time	Often	Most of the time
I keep in mind that proposed changes can affect the whole system.	❑	❑	❑	❑	❑
I think more than one or two people are needed to have success.	❑	❑	❑	❑	❑
I keep the mission and purpose of the organization in mind.	❑	❑	❑	❑	❑

I think small changes can produce important results.	❑	❑	❑	❑	❑
I consider how multiple changes affect each other.	❑	❑	❑	❑	❑
I think about how different people might be affected by the improvement.	❑	❑	❑	❑	❑
I try strategies that do not rely on people's memory.	❑	❑	❑	❑	❑
I recognize system problems are influenced by past events.	❑	❑	❑	❑	❑
I consider the past history and culture of the environment.	❑	❑	❑	❑	❑
I consider that the same action can have different effects over time, depending on the state of the system.	❑	❑	❑	❑	❑
